# *In Vitro* and *In Vivo* Biocompatibility Evaluation of Polyallylamine and Macromolecular Heparin Conjugates Modified Alginate Microbeads

**DOI:** 10.1038/s41598-017-11989-1

**Published:** 2017-09-15

**Authors:** Vijayaganapathy Vaithilingam, Bjørg Steinkjer, Liv Ryan, Rolf Larsson, Bernard Edward Tuch, Jose Oberholzer, Anne Mari Rokstad

**Affiliations:** 1 Materials Science and Engineering, Commonwealth Scientific and Industrial Research Organization (CSIRO), North Ryde, New South Wales Australia; 2 Centre of Molecular Inflammation Research and Department of Cancer Research and Molecular Medicine, Norwegian University of Science and Technology (NTNU), Trondheim, Norway; 3 Corline System AB, Uppsala, Sweden; 4 Department of Immunology, Genetics and Pathology, University of Uppsala, Uppsala, Sweden; 5 School of Medical Sciences, University of Sydney, Sydney, New South Wales Australia; 6 Department of Surgery, University of Illinois at Chicago, Chicago, Illinois United States of America; 7 The Central Norway Regional Health Authority (RHA), Trondheim, Norway

## Abstract

Host reactivity to biocompatible immunoisolation devices is a major challenge for cellular therapies, and a human screening model would be of great value. We designed new types of surface modified barium alginate microspheres, and evaluated their inflammatory properties using human whole blood, and the intraperitoneal response after three weeks in Wistar rats. Microspheres were modified using proprietary polyallylamine (PAV) and coupled with macromolecular heparin conjugates (Corline Heparin Conjugate, CHC). The PAV-CHC strategy resulted in uniform and stable coatings with increased anti-clot activity and low cytotoxicity. In human whole blood, PAV coating at high dose (100 µg/ml) induced elevated complement, leukocyte CD11b and inflammatory mediators, and in Wistar rats increased fibrotic overgrowth. Coating of high dose PAV with CHC significantly reduced these responses. Low dose PAV (10 µg/ml) ± CHC and unmodified alginate microbeads showed low responses. That the human whole blood inflammatory reactions paralleled the host response shows a link between inflammatory potential and initial fibrotic response. CHC possessed anti-inflammatory activity, but failed to improve overall biocompatibility. We conclude that the human whole blood assay is an efficient first-phase screening model for inflammation, and a guiding tool in development of new generation microspheres for cell encapsulation therapy.

## Introduction

Microencapsulating pancreatic islets is a strategy explored as a potential cellular therapy for type 1 diabetes to overcome immune rejection without toxic immunosuppression. The semi-permeable membrane of alginate microspheres allows the diffusion of nutrients, oxygen and metabolites essential for cell survival and function but also provides immunoprotection by preventing the entry of immune cells and large molecular weight antibodies^1^. The microspheres have been made of alginate gelled with divalent cations (Ca^2+^ or Ba^2+^) and often stabilised by layer-by-layer (LBL) deposition of polycation such as poly-L-lysine (PLL) to reduce permeability and improve mechanical strength. Encapsulated islets have shown great promise to normalize blood glucose levels and achieve euglycaemia in varied preclinical models of allo- and xeno- transplantation^2^. However, long-term graft survival is limited and varied considerably mostly due to pericapsular fibrotic overgrowth (PFO)^3^.

Despite several years of research employing different strategies^4–11^, a truly biocompatible microsphere devoid of PFO upon implantation is hard to develop. PFO is a complex process and involves various factors such as protein adsorption, leukocyte activation and granulation formation consisting of fibroblasts and macrophages^12^. Recently it was also evident that complement is involved in the host response^13^. The immediate inflammatory responses are possible to study *in vitro* using a human whole blood model containing proteins and cells capable of being activated^14^. In order to determine a long-term biocompatibility outcome however, testing is best carried out *in vivo*. There are no simple approaches to determining well-functioning devices, and the outcomes often depend on the animal model^15^. Also, the various alginate microspheres which showed promise in the preclinical setting, failed to make a significant impact in the clinic^16,17^. The findings in the clinical setting are also complicated with the encapsulated pancreatic islets potentially releasing inflammatory and immunogenic components. To date there are no clinical studies evaluating the biocompatibility of alginate microspheres alone due to ethical concerns. The human whole blood model encompasses physiological qualities allowing cross-talk between proteins and leukocytes, and has proven efficient in evaluating inflammatory properties^15^. Although there is correspondence between the inflammatory outcome towards alginate microspheres in whole blood and the fibrotic outcome as described in several small animal studies^15^, the ultimate study of direct comparisons of the same material has not yet been described. In summary, there is a need for determining the usefulness of the *in vitro* whole blood model compared to *in vivo* outcomes when examining the suitability of promising candidate devices.

Surface modification with heparin has been previously studied with conflicting results^18,19^. We showed previously that the binding of heparin to alginate microbeads via avidin improved the biocompatibility and reduced PFO, but the heparin activity was gradually lost over time^18^. In the present study, we therefore investigated the usefulness of a proprietary polycationic linker, polyallylamine (PAV) previously used to produce polyelectrolyte multilayer coatings on alginate substrates^20^ and cross-linked alginate microbeads with enhanced mechanical strength and long-term stability^21^. PAV was used as a template for LBL coating of macromolecular Corline Heparin Conjugates (CHC) onto the surface of barium alginate microbeads. CHC is a water soluble macromolecular heparin conjugate where each conjugate has 70 heparin molecules bound covalently to an extended aliphatic polyamine carrier chain thereby exposing the anti-thrombin binding sites of heparin^22^.

The current study presents a strategy for modification of barium alginate microbeads with PAV and CHC, and furthers on the evaluation of the inflammatory potential in the human whole blood model and the fibrotic outcome in an immune competent Wistar rat model. This is the first study to directly compare the outcomes in a human whole blood model with the outcome in an intraperitoneal rodent transplantation model.

## Results

### Binding of CHC to alginate microbeads via cationic linkers

**Figure 1:**
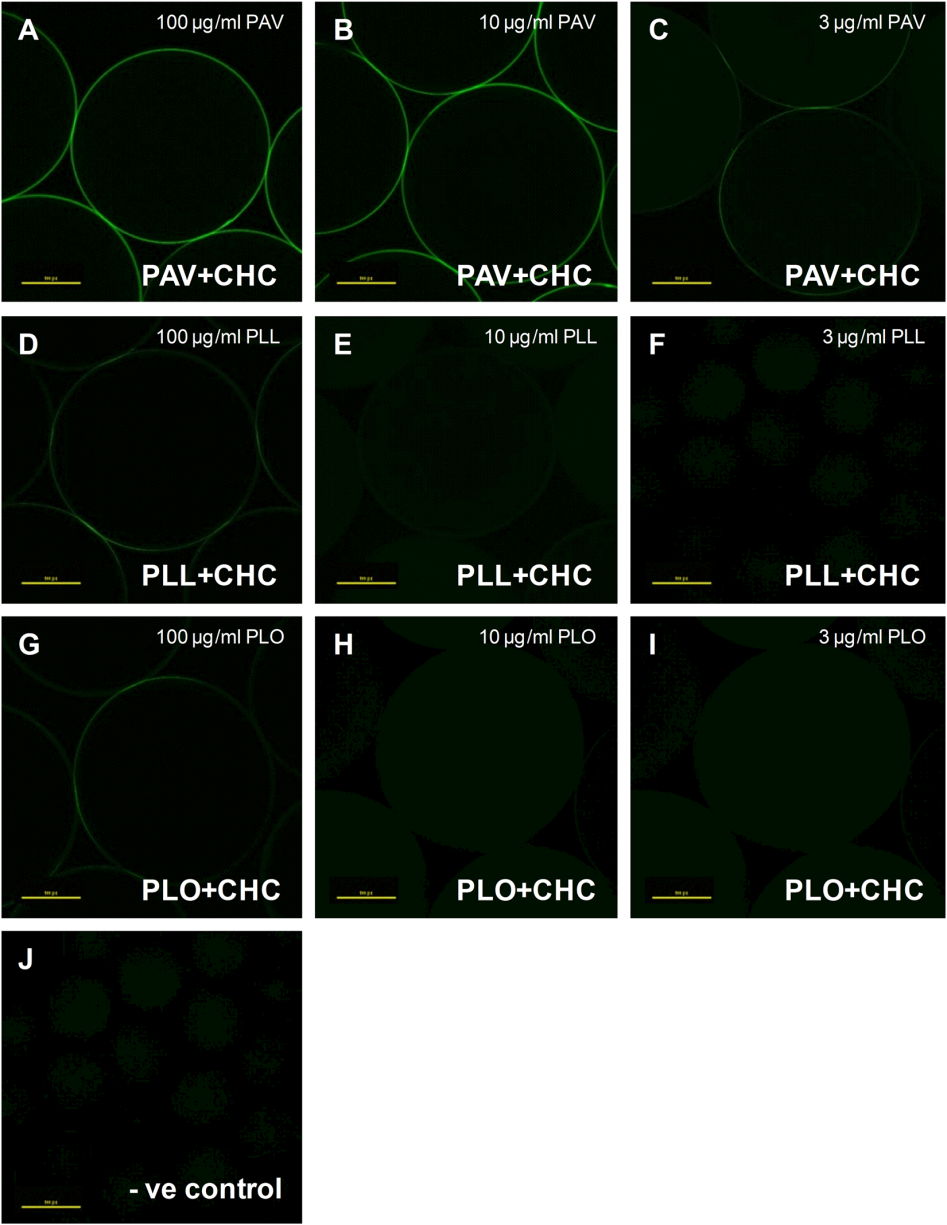
LBL binding of CHC to alginate microbeads by cationic linkers. Representative confocal images of fluorescently labelled CHC (36 µg/ml) binding to alginate microbeads via polycationic linkers PAV, PLL and PLO at varied concentrations of 100 µg/ml (**A**,**D**,**G**), 10 µg/ml (**B**,**E**,**H**) and 3 µg/ml (**C**,**F**,**I**). Negative control (**J**) is CHC added to alginate microbeads without linkers (Number of separate experiments was 3, and number of replicates in each group was 3).

The potential of the three cationic linkers to bind CHC to alginate microbeads was evaluated using confocal laser scanning microscopy (CLSM). The average size of the microbeads used in this study was 736 ± 4.2 µm (range 664.3–799.8 µm, median 735.3 µm). PAV, PLL and poly-L-lysine (PLO) (100, 10 & 3 µg/ml) were used to double coat alginate microbeads with fluorescently labelled CHC-Alexa488 using the LBL approach. CLSM images demonstrated that using PAV at both 100 and 10 µg/ml, a uniform coating of CHC could be established on the surface of alginate microbeads (Fig. 1A and B). PAV at 3 µg/ml resulted in a weak CHC binding with the lack of uniform heparin coating as evident from the incoherent fluorescent signal (Fig. 1C). Coating of alginate microbeads using PLL or PLO did not result in efficient CHC binding at a high concentration of 100 µg/ml (Fig. 1D and G) and no binding at all at low concentrations of 10 µg/ml (Fig. 1E and H) and 3 µg/ml (Fig. 1F and I) respectively. These results suggest the polycationic linker PAV as a useful primer to bind CHC efficiently to alginate microbeads by the LBL method. Further experiments were carried out only with PAV-CHC coated alginate microbeads. Hereafter, LBL modified micobeads containing high (100 µg/ml) and low (10 µg/ml) concentrations of PAV without CHC will be referred to as PAV_(high)_ and PAV_(low)_ respectively and those with CHC will be referred as PAV_(high)_ + CHC and PAV_(low)_ + CHC.

**Figure 2:**
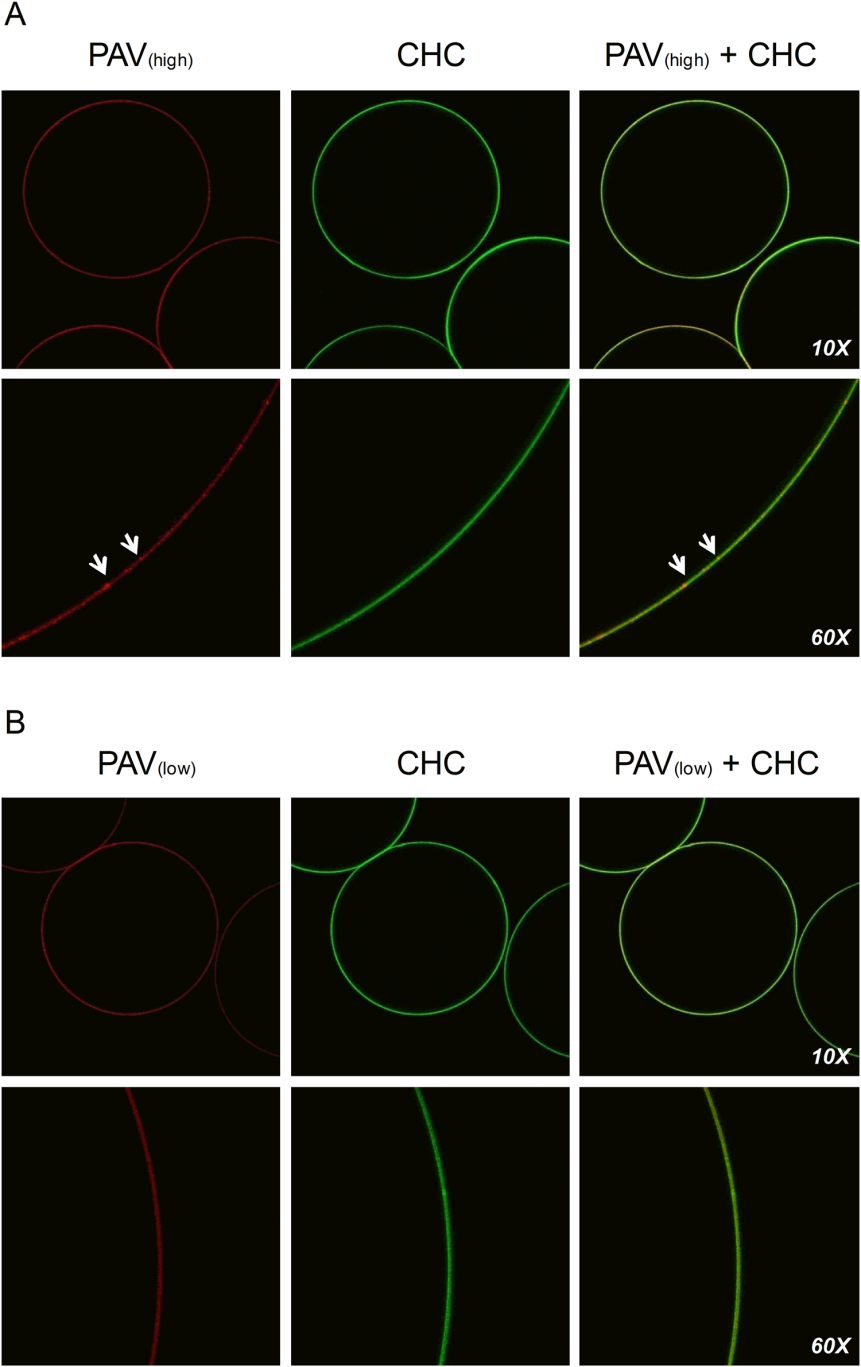
Confocal imaging of LBL modified microbeads. Representative confocal images of LBL modified alginate microbeads containing high (100 µg/ml) or low (10 µg/ml) concentrations of fluorescently labelled PAV (PAV-Cy5.5; red) and 36 µg/ml CHC (CHC-Alexa488; green) cultured for 1, 7, 14 and 21 days in phosphate buffered saline post-heparinization (n = 3 for each group). The figures above are representative confocal images of LBL modified beads taken at day 21 post-heparinization. Arrows point to intermittent areas of excess deposition of PAV which are not completely masked by CHC (seen in higher magnification images of PAV_(high)_ + CHC microbeads) and hence remain exposed to initiate an immune response.

### Stability of PAV and CHC coatings on alginate microbeads

The long-term stability of PAV and CHC coatings was evaluated by CLSM using fluorescently labelled PAV-Cy5 and CHC-Alexa 488. PAV and CHC coatings in both PAV_(high)_ + CHC and PAV_(low)_ + CHC microbeads were stable with no signs of evident leaching for up to 21 days when kept in phosphate buffered saline (PBS) and at 37 °C (Fig. 2A and B) as compared to the coating at day 1 (Supplementary Figure 1A and B) suggesting a strong interaction of PAV and CHC to alginate. However, at higher magnification the high concentration PAV coatings (100 µg/ml) led to intermittent areas of excess deposition of PAV being exposed (as denoted by arrows) as opposed to those generated with low PAV concentration of 10 µg/ml (Fig. 2A and Supplementary Figure 1A).

**Figure 3:**
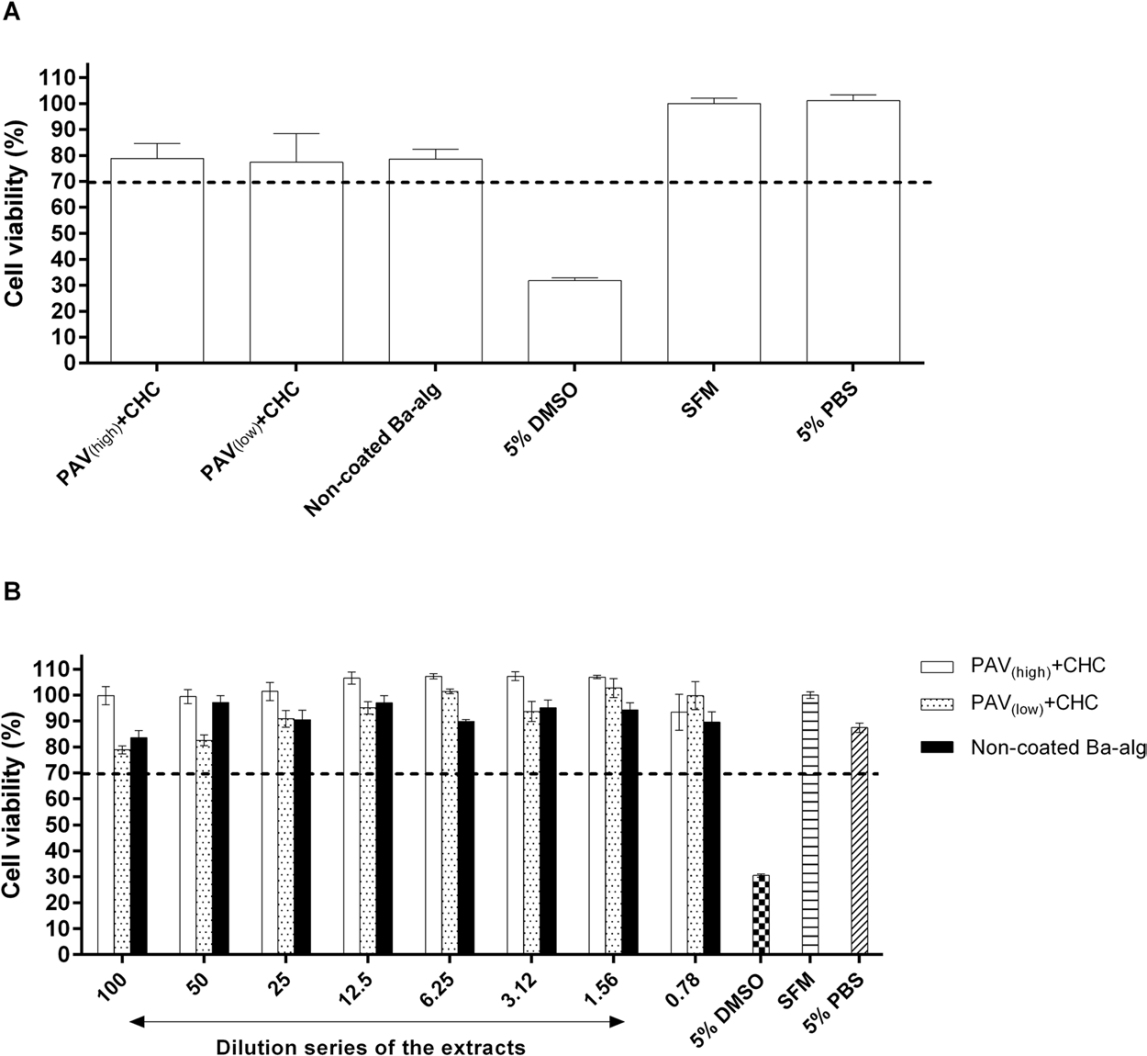
Cytotoxicity of LBL modified microbeads. Direct contact (**A**) and indirect extract (**B**) cytotoxicity assay of PAV_(high)_ + CHC and PAV_(low)_ + CHC microbeads compared to non-coated barium alginate microbeads using mouse fibroblast L929 cells after 24 hr in culture. A cut-off value of <70% was considered cytotoxic as seen with the positive control 5% DMSO and a value >70% was considered non-toxic as seen with the negative control 5% PBS and SFM reference control. Values = mean ± SEM (n = 3 for each group).

### Anti-clotting activity of bound CHC

To determine the bioactivity of CHC bound to alginate microbeads via PAV_(high)_ or PAV_(low)_ linker, an APTT assay was carried out and the clotting times measured. Clotting times for both PAV_(high)_ + CHC and PAV_(low)_ + CHC microbeads were significantly higher at days 1, 7, 14 and 21 post-heparinization compared to non-coated microbeads and plasma controls (Table 1). There was no significant difference in the clotting times at different time points between PAV_(high)_ + CHC and PAV_(low)_ + CHC microbeads. On the other hand, a prolonged clotting time of >170 s was measured when CHC was added directly to plasma suggesting the strong bioactivity of the macromolecular heparin conjugate. This study shows that heparin activity is retained in the treated microbeads for at least three weeks of culture.

**Table 1:** Anti-clotting activity of bound CHC measured over 21 days post-heparinization.

Groups	Day 1 post-heparinization (sec)	Day 7 post-heparinization (sec)	Day 14 post-heparinization (sec)	Day 21 post-heparinization (sec)
Plasma alone	33.6 ± 0.5	33.9 ± 0.8	33.3 ± 0.9	34.6 ± 0.7
Non-coated Ba-alg microbeads	52.7 ± 1.6	51.4 ± 2.1	48.1 ± 2.7	50.6 ± 1.7
PAV_(high) _+CHC microbeads	130.1 ± 19.9*	133.3 ± 18.7*	142.3 ± 16*	138.3 ± 16.9*
PAV_(low)_ + CHC microbeads	141 ± 14.8*	135.8 ± 18.4*	138.6 ± 19.5*	129.7 ± 19.2*
Plasma + CHC	>170	>170	>170	>170

APTT test was carried to determine the long term bioactivity of CHC (1 mg/ml) bound to alginate microbeads via high (100 µg/ml) or low (10 µg/ml) concentrations of PAV. Values = mean ± SEM (n = 3 for each time point and group); *p < 0.001 for clotting times (in sec) at days 1, 7, 14 & 21: PAV_(high)_ + CHC and PAV_(low)_ + CHC microbeads > plasma and non-coated Ba-alg microbeads (ANOVA with *posthoc* Duncan’s Multiple-Comparison test). Upper limit of the assay is 170 sec.

### Cytotoxicity testing of PAV-CHC coated alginate microbeads

The toxicity of PAV-CHC coated alginate microbeads was determined using a direct contact cytotoxicity assay. For this assay, PAV_(high)_ + CHC and PAV_(low)_ + CHC microbeads were added directly to form a monolayer on top of L929 cells and incubated for 24 hr before analysis. Neither PAV_(high)_ + CHC nor PAV_(low)_ + CHC microbeads elicited a cytotoxic response from the L929 cells with cell viabilities similar to non-coated alginate microbeads (Fig. 3A). However, the unbound soluble forms of the cationic linkers polyallylamine (PAV), poly-L-lysine (PLL) and poly-L-ornithine (PLO) were cytotoxic (Supplementary Figures 1 and 3; Appendix S1), while the Corline Heparin Conjugate (CHC) was non-toxic in a concentrations up to 500 µg/ml (Supplementary Figures 2 and 3; Appendix S1).The cytotoxicity of leaking products was measured indirectly by incubating the microbead supernatants with L929 cells. None of the extracts from either PAV_(high)_ + CHC or PAV_(low)_ + CHC microbeads elicited a cytotoxic response from the L929 cells, with cell viabilities well above the cut-off value of 70% for all dilutions tested (Fig. 3B).The data suggest that PAV-CHC coatings were stable without leaching for at least 24 hr in culture.

### *In vitro* biocompatibility evaluation of PAV-CHC coated alginate microbeads

The inflammatory potential of PAV, PAV-CHC coated microbeads as well as non-coated microbeads was evaluated in human whole blood by measuring levels of terminal complement complex (TCC), a marker of complement activation; leukocyte CD11b expression; and selected inflammatory cytokines.

#### TCC

**Figure 4:**
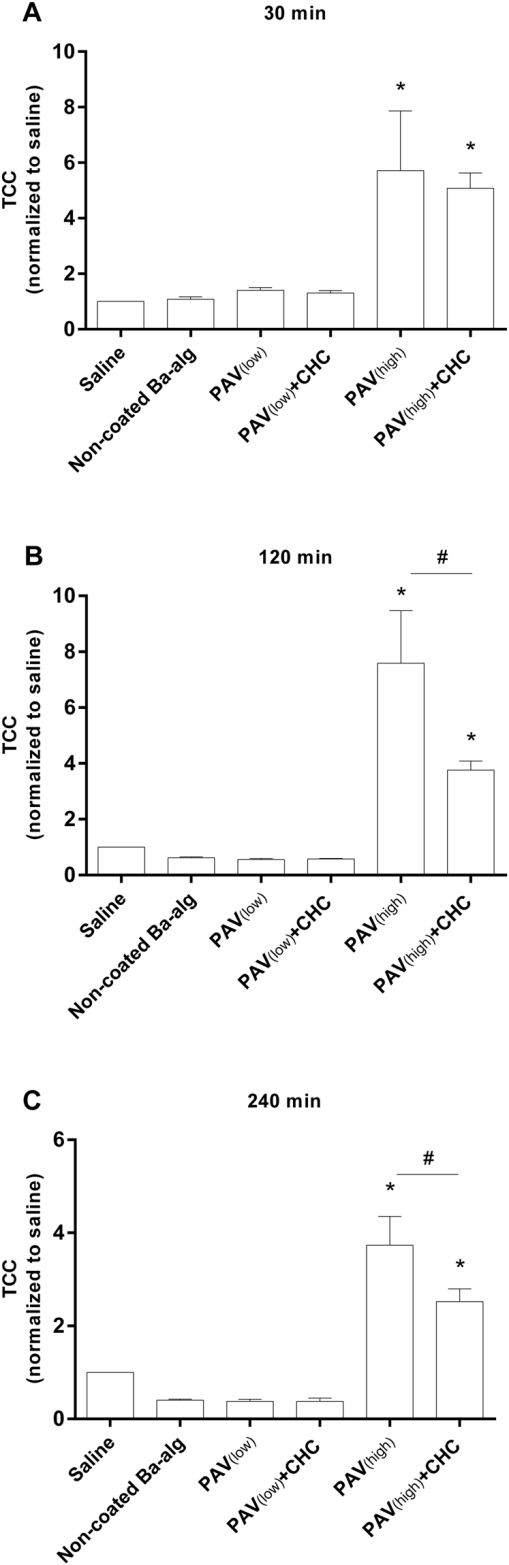
Effect of LBL modified microbeads on complement activation (Terminal Complement Complex [TCC]). Relative TCC formation compared to saline after incubation in whole blood with various coated and non-coated alginate microbeads for 30 (**A**), 120 (**B**) and 240 (**C**) min. Values are mean ± SEM (n = 4–5 of separate experiments with different donors); *p < 0.0001 for microbeads versus saline control (ANOVA, with *posthoc* Duncan’s Multiple-Comparison test) and ^#^p < 0.0001 between PAV_(high)_ and PAV_(high)_ + CHC (Student’s t-test). Values (AU/ml) are normalized to saline based on data obtained from two separate experiments with different donors each study, given in Supplementary Figure 4.

Figure 4 show the TCC values relative to the saline control for the various additives (non-coated, PAV_(low)_, PAV_(low)_ + CHC, PAV_(high)_ and PAV_(high)_ + CHC microbeads). The amount of TCC for the non-coated, PAV_(low)_ and PAV_(low)_ + CHC microbeads was low and not significantly different from the saline control after 30 min incubation (Fig. 4A), and gradually became lower than the saline control with incubation time (Fig. 4B and C). The coatings of PAV_(high)_ and PAV_(high)_ + CHC induced a significant increase in TCC as compared to the saline control. At 120 and 240 min, the TCC formation was significantly higher for PAV_(high)_ compared to the PAV_(high)_ + CHC suggesting the complement activation potential of unmasked PAV_(high)_ (Fig. 4B and C). The TCC arbitrary unit’s time-kinetics are given in Supplementary Figure 4A and B including the positive control zymosan. Supplementary Figure 4A and B show an overall increase in TCC by incubation time, which is due to the combination of the background activation by the polypropylene vials in combination with the surface activating potential of the various additives.

#### Leucocytes CD11b expression

**Figure 5:**
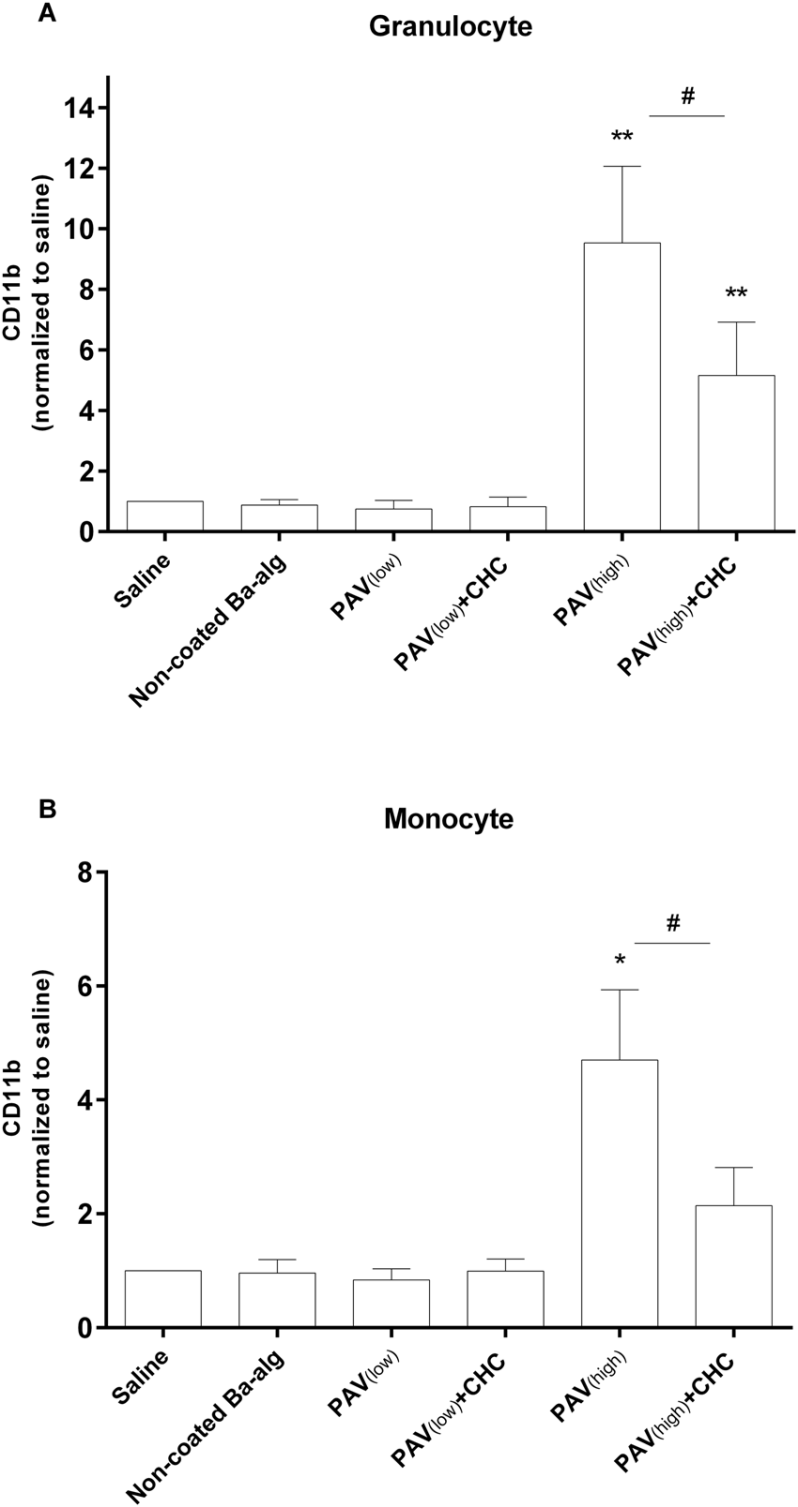
Effect of LBL modified microbeads on leucocyte activation. Leucocyte activation as measured by CD11b expression on both granulocytes (**A**) and monocytes (**B**) relative to saline after incubation of whole blood with various coated and non-coated alginate microbeads for 240 min. Values are mean ± SEM (n = 3 of separate experiments with different donors); **p < 0.0001 and *p < 0.001 for PAV_(high)_ and PAV_(high)_ + CHC versus the saline control (ANOVA, with *posthoc* Duncan’s Multiple-Comparison test), and ^#^p < 0.0001 and <0.001 between PAV_(high)_ and PAV_(high)_ + CHC for granulocytes and monocytes respectively (Student’s t-test). Values (mean fluorescence intensity; MFI) are normalized to saline from data of two different experiments with different donors. MFI values from the two separate experiments are shown in Supplementary Figure 5.

The expression of CD11b on granulocytes and monocytes is shown in Fig. 5. Both the addition of PAV_(high)_ and PAV_(high)_ + CHC microspheres induced significantly higher CD11b expression in granulocytes compared to the saline control (Fig. 5A). PAV_(high)_ significantly induced CD11b expression in monocytes, and in general followed the same pattern as seen for the granulocytes (Fig. 5B). A reduced CD11b expression was obtained by PAV_(high)_ + CHC, which indicates that the heparin coating has the potential to mask the stimulatory effect of PAV_(high)_. The non-coated alginate microbeads, as well as the PAV_(low)_ and PAV_(low)_ + CHC was at comparable level as the saline control. Mean fluorescence intensity (MFI) values are given in Supplementary Figure 5.

#### Cytokines

**Figure 6:**
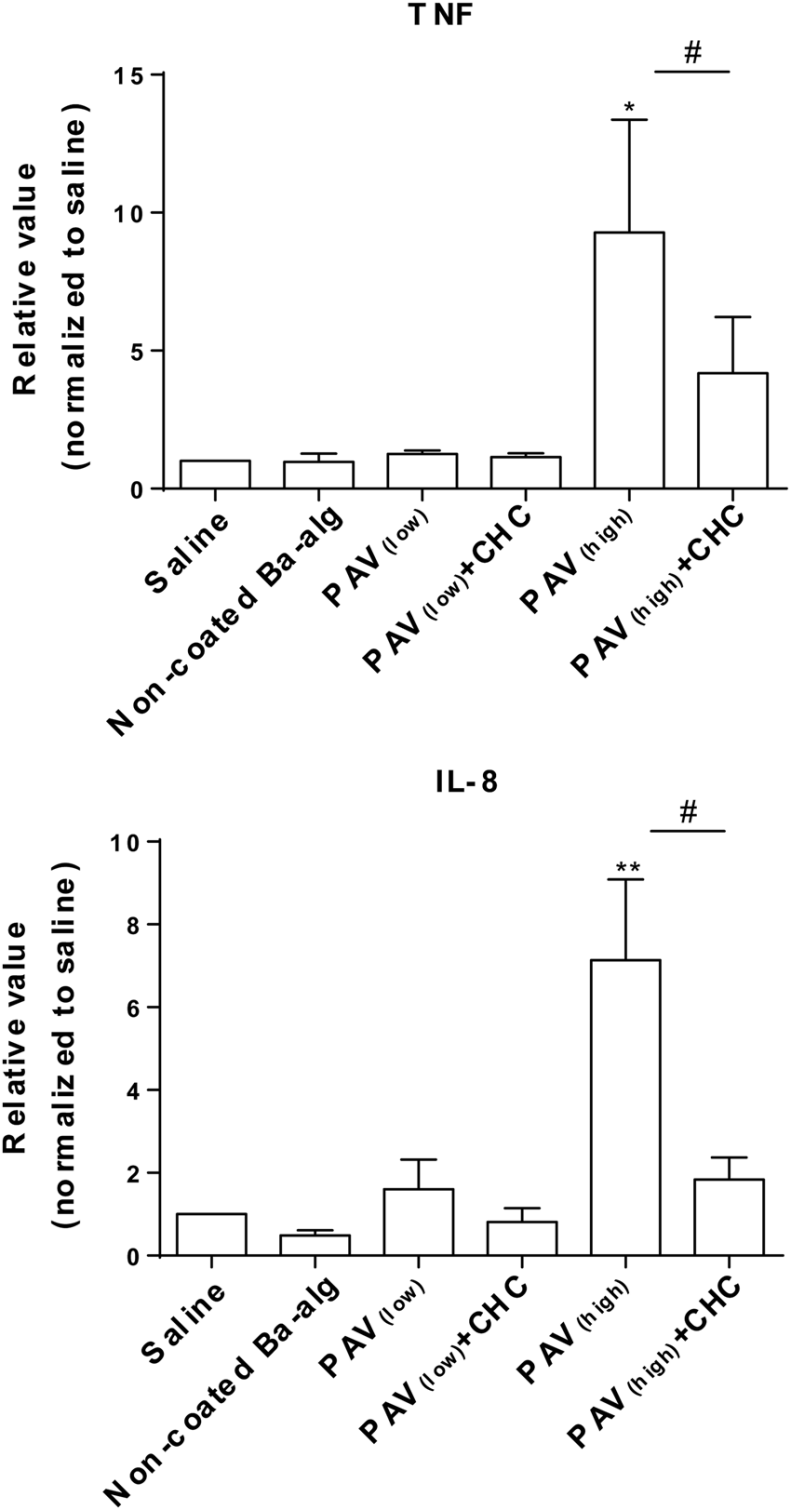
Effect of LBL modified microbeads on TNF-α and IL-8 response. PAV _(high)_, PAV_(high)_ + CHC, PAV _(low)_, PAV_(low)_ + CHC and non-coated Ba-alg microbeads incubated in lepirudin anti-coagulated whole blood for 240 min. Values are mean ± SEM (n = 4–5 of separate experiments with different donors); *p < 0.01 and **p < 0.0001 for microbeads versus saline control (ANOVA, with *posthoc* Duncan’s Multiple-Comparison test) and ^#^p < 0.05 between PAV_(high)_ and PAV_(high)_ + CHC (Student’s t-test). Values (measured as pg/ml) are normalized to saline and measured in plasma obtained from four to five independent donors. Raw data values for plasma baseline, saline, LBL modified microbeads and the positive control zymosan measured are shown in Supplementary Figure 6.

**Figure 7:**
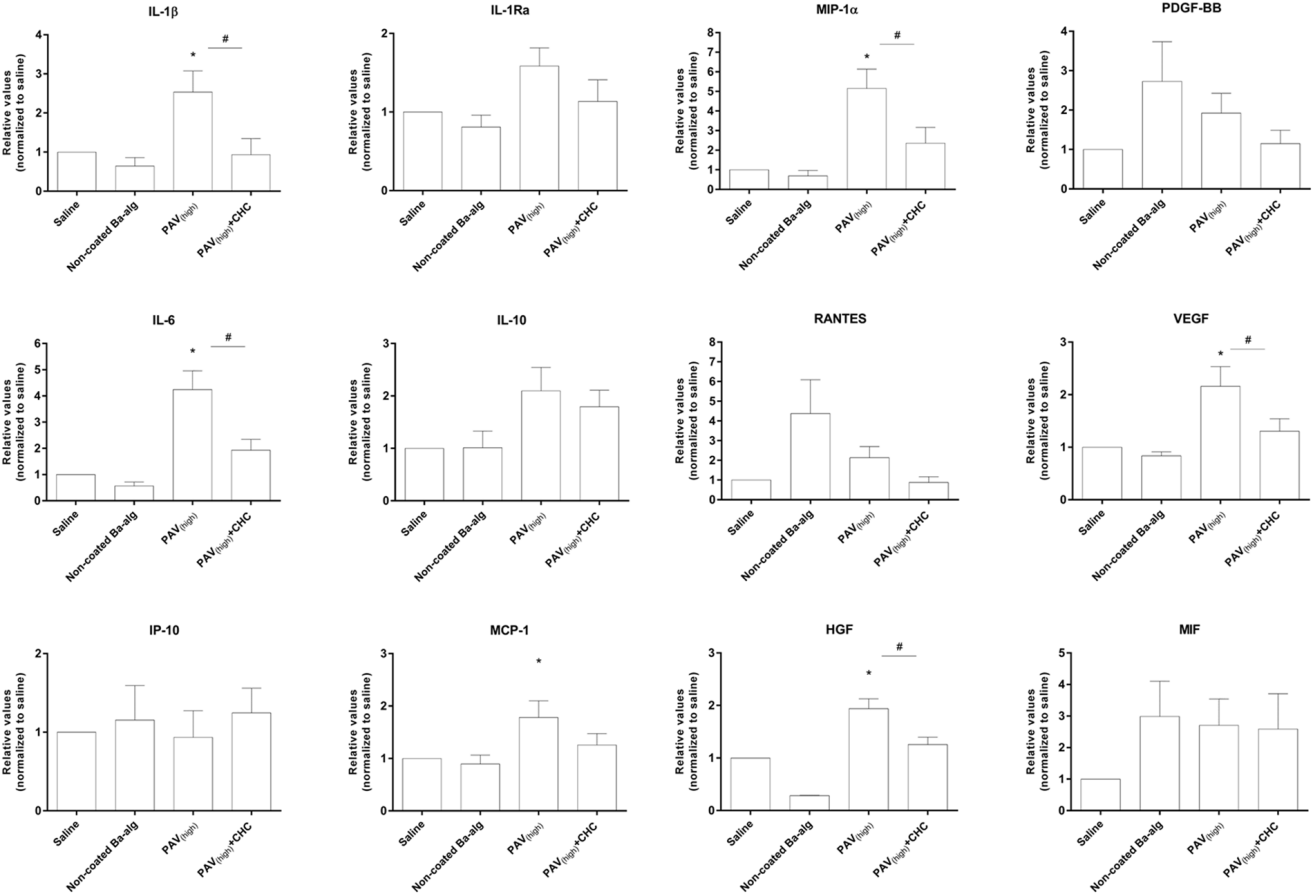
Effect of LBL modified microbeads containing PAV_(high)_ on the cytokine response. PAV _(high)_, PAV_(high)_ + CHC or non-coated Ba-alg microbeads incubated in lepirudin anti-coagulated whole blood for 240 min. Values are mean ± SEM (n = 5); *p < 0.05 for PAV_(high)_ > saline, non-coated, PAV_(high)_ + CHC microbeads and ^#^p < 0.05 for PAV_(high)_ > PAV_(high)_ + CHC (ANOVA with *posthoc* Duncan’s Multiple-Comparison test). Values (measured as pg/ml) are normalized to saline and measured in plasma obtained from five independent donors. Raw data values for plasma baseline, saline, LBL modified microbeads and the positive control zymosan measured are shown in Supplementary Figure 7.

The cytokine/chemokine inducing potential of the various coated and non-coated alginate microbeads was compared by use of TNF and IL-8 (Fig. 6). These inflammatory cytokines have previously been shown to be reliable and consistent markers of the inflammatory potential of microspheres^23,24^. PAV_(high)_ significantly induced TNF and IL-8, while in comparison a significant reduction was found by PAV_(high)_ + CHC, suggesting a masking potential of CHC (Fig. 6A). In contrary, PAV_(low)_, PAV_(low)_ + CHC and non-coated barium alginate microbeads did not induce significant different values of TNF and IL-8 from the saline control (Fig. 6B). A panel of other inflammatory mediators were also evaluated for the PAV_(high)_ and PAV_(high)_ + CHC microbeads (Fig. 7). The PAV_(high)_ significantly induced the expression of proinflammatory cytokines IL-1β and IL-6, chemokines MIP-1α and MCP-1 and the growth factors VEGF and HGF. Coating of CHC to PAV_(high)_ significantly diminished the induction of IL-1β, IL-6, MIP-1α, VEGF and HGF, and also reduced theMCP-1 levels although significant difference were not achieved (Fig. 7). Non-coated microbeads were inert and induced comparable secretion of IL-1β, IL-6, MIP-1α and MCP-1 as the saline control (Fig. 7). Addition of PAV_(high)_ had no effect on IP-10, RANTES and PDGF-BB induction, while a non-significant elevation of RANTES and PDGF-BB levels by the non-coated microbeads was detected (Fig. 7). Supplementary Figures (6 and 7) are showing the cytokine values in pg/ml. Overall, these data suggest that PAV_(high)_ but not PAV_(low)_ containing microbeads had the ability to induce inflammation, and further that the CHC coating could reduce the inflammatory potential although not reduce it below non-coated barium microbeads.

### *In vivo* biocompatibility evaluation of LBL modified microbeads

**Figure 8:**
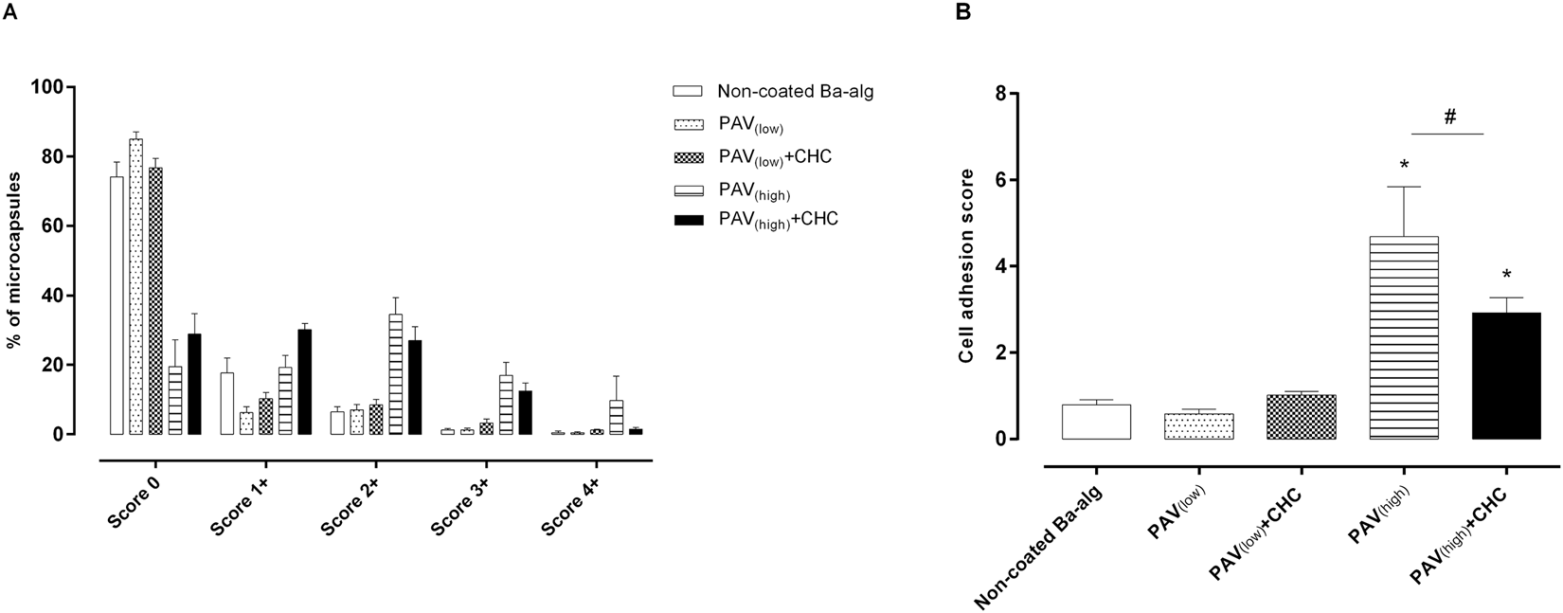
Assessment of PFO on retrieved LBL modified microbeads. The fibrotic scores (**A**) and cell adhesion scores (**B**) on retrieved microbeads transplanted into peritoneal cavity of Wistar rats. Extent of host cell adhesion on retrieved LBL modified microbeads is represented by a cell adhesion score, on a scale of 0 (no cell adhesion) to 16 (complete host cell adhesion). Values are mean ± SEM (n = 4–8 animals per group); *p < 0.0001 for cell adhesion score where PAV_(high)_and PAV_(high)_ + CHC > saline, non-coated, PAV_(low)_ and PAV_(low)_ + CHC microbeads and ^#^p < 0.0001 for cell adhesion score where PAV_(high)_ > PAV_(high)_ + CHC microbeads (ANOVA with *posthoc* Duncan’s Multiple-Comparison test).

Non-coated, PAV_(low)_, PAV_(low)_ + CHC, PAV_(high)_ and PAV_(high)_ + CHC microbeads were transplanted into the peritoneal cavity of Wistar rats for three weeks and assessed for fibrotic overgrowth using a scoring system. The retrieval rate was approximately equal between the non-coated, PAV_(low)_ and PAV_(low)_ + CHC microbead, but reduced in the groups transplanted with PAV_(high)_ and PAV_(high)_ + CHC microbeads (Table 2). The fibrotic score of PAV_(high)_ was distributed as 2 + (~34.5%), 3 + (~17%) and 4 + (~9.8%) with some clumped and adherent to the abdominal organs. Addition of CHC to PAV_(high)_ resulted in significantly less fibrosis with the majority of microbeads free floating and only a few adherent to abdominal organs. The fibrotic score of the retrieved PAV_(high)_ + CHC was distributed as 0 (~28.8%), 1 + (~30.1%), 2 + (~27%) or 3 + (~12.5%), and the percentage with score of 4 + (~1.5%) was significantly less than for PAV_(high)_ (Fig. 8A). The extent of PFO as measured by the cell adhesion score was also significantly reduced by ~1.6 fold in the PAV_(high)_ + CHC compared to the PAV_(high)_ (2.9 ± 0.4 vs 4.7 ± 1.2) (Fig. 8B). Despite the slight reduction in PFO, both PAV_(high)_ and PAV_(high)_ + CHC microbeads had significant amount of PFO compared to non-coated microbeads suggesting that PAV_(high)_ containing microbeads were not biocompatible. On the contrary, the PAV_(low)_ and PAV_(low)_ + CHC coated microbeads were mainly free floating and majority had a fibrotic scores of <2 + (PAV_(low)_: ~98% vs PAV_(low)_ + CHC: ~95%) similar to non-coated microbeads (fibrotic score of <2 + : ~97%) (Fig. 8A). Both PAV_(low)_ and PAV_(low)_ + CHC coated microbeads had low cell adhesion scores of ~0.6 and ~1.02 respectively similar to non-coated microbeads with a fibrotic score of ~0.8 suggesting their biocompatible nature (Fig. 8B). These data indicate that PAV_(high)_ containing microbeads had the ability to induce fibrotic overgrowth, in consistent with the inflammatory potential and leukocyte activation found in the *in vitro* whole blood assay.

**Table 2:** Morphological assessment of LBL modified microbeads retrieved from the peritoneal cavity of immunocompetent rats in different implantation settings.

Groups	Graft recovery (%)	Intact microbeads (%)	State of retrieved microbeads
**A. Implantation of empty microbeads**			
Non-coated Ba-alg (n = 8)	88.3 ± 2.4	100	FF
**B. Implantation of PAA**_**(low)**_ **coated microbeads**			
PAV_(low)_ alone (n = 4)	87.5 ± 1.7	100	FF
PAV_(low)_ + CHC (n = 4)	89.0 ± 1.3	100	FF
**C. Implantation of PAA**_**(high)**_ **coated microbeads**			
PAV_(high)_ alone (n = 4)	78.5 ± 1.5	100	FF, some A & C
PAV_(high)_ + CHC (n = 8)	82.3 ± 2.0	100	FF & few A

FF – Freely floating in the peritoneal cavity; A – Adherent to abdominal organs; C – Clumping of microbeads.

## Discussion

There is a critical need for the development of novel biocompatible encapsulation devices as well as an *in vitro* experimental model serving as a predictor of the host reactivity. Our study addressed the above issues by new microspheres design using PAV and CHC, secondly determining the inflammatory potential using a human whole blood model, and finally verifying to the host response in an immunocompetent rat model. The new coating strategy did not succeed in terms of improving the biocompatibility, but we found a connection between the inflammatory responses of complement TCC, leukocyte activation and inflammatory cytokines/chemokine induction; and the host reaction after three weeks intraperitoneal implantation. Our data thus points to a link between the inflammatory potential and the fibrotic response against microbeads in the initial post-implantation phase. In summary, this study shows that the whole blood assay is a useful tool for assessing the likelihood of major reactivity to devices when implanted *in vivo*, and facilitates *in vitro* assessment of engineering interventions designed to reduce the host response to these devices. Our strategy also comprised the testing of cross-linkers for CHC modification including stability and toxicity testings. In the next sections the findings are discussed chronologically with our testing strategy.

LBL heparin coating suggested that only PAV but not PLL or PLO can bind CHC efficiently to alginate microbeads. Both PAV_(high)_ and PAV_(low)_ established a complete and uniform coating of CHC on the surface of alginate microbeads as demonstrated by confocal microscopy. The polycation, PAV, is a weak base containing a high density of primary amino groups regularly spaced along its polymeric hydrocarbon backbone and existing as free amine at neutral pH^25,26^. The high binding capacity of PAV might be attributed to the high cationic charge density due to the primary amino groups and the short hydrocarbon side chain compared to PLL or PLO^27^. Confocal microscopy further confirmed the stability of PAV and CHC coatings and the bound CHC retained its anti-clotting activity for at least three weeks as opposed to CHC bound via avidin cross-linker. Cross-linking CHC to alginate microbeads via avidin resulted in a gradual loss of the heparin biological activity over time as demonstrated in our previous study^28^. A similar outcome was seen when CHC was anchored to the surface of primary islets incorporating avidin to prevent instant blood-mediated inflammatory reaction (IBMIR) with complete loss of heparin coating at four weeks post-implantation^29^. The loss of heparin activity seen with avidin but not PAV might be attributed to the binding configuration of CHC to alginate substrates. It has been demonstrated that heparin activity is lost if they are linked to its substrate by multiple bonds and that immobilized heparin must be linked only by a single point of attachment to retain its biological activity^22,30^. Accordingly, it was shown that heparin surfaces generated by random cross-linking techniques resulted in heparin molecules being bound to substrate by multiple bonds compared to CHC binding via amines which resulted in single-point attachment and hence substantially increased heparin activity^22^. Similarly, in this study binding CHC via the amine PAV might have taken the single-point attachment configuration thereby providing a flexible layer of heparin molecules with exposed anti-thrombin binding sites and hence retaining its anti-clotting activity over time. Further, binding CHC to alginate microbeads should not have altered the permeability of the microbeads as demonstrated in our previous study^28^. To our knowledge, this is the first study investigating the binding of the unique macromolecular CHC to barium alginate microbeads via the cationic linker PAV.

The non-toxic nature of CHC has been well established and might be due to the fact that all the cross-linking impurities have been carefully eliminated prior to use^31^. Consistant with this is that CHC was found not to be cytotoxic at the high concentration of 0.1% v/v. Among the three cationic linkers tested, PAV was more cytotoxic than PLL and PLO. This is consistant with the toxicity profiles on primary islets comparing PLL and PAV^32^. It has been shown previously that the toxicity of free polycations vanishes when they are bound in a polymer complex^33^. Similarly, in this study we found that the toxicity of free PAV disappeared when they were bound to alginate microbeads and cross-linked with CHC as demonstrated with the non-toxic nature of PAV_(high)_ + CHC and PAV_(low)_ + CHC microbeads in the direct contact cytotoxicity assay. Further, the non-toxic milieu of culture supernatants containing PAV-CHC complexed alginate microbeads in indirect extract cytotoxicity study suggest that the PAV-CHC coatings were stable and were not leaching into the culture media.

Having established that functional CHC can be bound efficiently to alginate microbeads via PAV, we then tested the biocompatibility of PAV-CHC complexed alginate microbeads both *in vitro* (human whole blood model) and *in vivo* (rodent model). The lepirudin based human whole blood assay preserves all the complement proteins and the ability of the leucocytes to be activated, thus making it possible to assess the inflammatory potential of surface modified alginate microbeads^14^. Previously it has been used to study blood compatibility of perfusion biomaterials^34–36^, biosensors^37,38^ and more recently PLL and PMCG alginate microbeads^23,39,40^. In the present study PAV_(high)_ was found to provoke inflammation due to the increased TCC, leukocyte CD11b expression and cytokines, and was significantly more potent as compared to PAV_(low)_ and non-coated barium alginate microbeads. The complement activating potential of the PAV_(high)_ containing microbeads might be attributed to the exposed nucleophilic amino groups which are known to be potent activators of the alternative complement pathway^41^ in a similar way as observed for PLL^24,40^ and PLO^24^ coated microspheres. Heparin is known to be interacting with several of the proteins of the complement cascade^42^ and to inhibit the complement reactivity^39^. The reduced complement activation as seen by PAV_(high)_ + CHC microbeads is likely to involve the anti-complement effect of the heparin coating. The binding configuration of CHC to PAV layer as seen previously with surface modified polymethylmethacrylate polymers using end-point heparin attachments might also be advantageous by preserving anti-complement effects of the heparin coating^43^. In addition, there is a possibility of the heparin coating to cause a direct neutralising effect on the PAV reactivity. Although, confocal microscopy demonstrated an uniform distribution of CHC on the surface of PAV_(high)_ microbeads, the degree of interaction may vary resulting in exposure of PAV. Inadequate neutralisation of PAV by the heparin coating could explain the complement activation properties of the PAV_(high)_ + CHC microbeads.

Complement activation plays a central role in leukocyte potentiation, and the activating product C5a seems to play an important role for activating the leukocytes to express CD11b^39^. In addition, the surface opsonisation by complement activating products as C3b/iC3b further serves as ligands for cellular attachment through the activated CD11b/CD18^24,44^. In fact, we previously demonstrated that polycation containing microspheres induced inflammatory cytokines by the involvement of activated complement C5a, C3b/iC3b opsonisation and cell-adhesion through CD11b/CD18^24^. In this study, we found that PAV_(high)_ containing microbeads increased the expression of CD11b, and the PAV_(high)_ + CHC coated microbeads reduced the CD11b leukocyte expression. The PAV_(high)_ microbeads significantly enhanced the secretion of the pro-inflammatory cytokines (TNF-α, IL-1β and IL-6), pro-inflammatory chemokines (IL-8, MIP-1α and MCP-1) and growth factors (VEGF and HGF), while the additional coating with CHC reduced the induction of pro-inflammatory cytokines/chemokines in consistent with the complement activation and the CD11b expression profiles. Overall, these findings indicate that CHC have a potential as a non-inflammatory coat given an alternative coating method. The PAV_(low)_, PAV_(low)_ + CHC were low inflammatory inductors at corresponding levels as the non-coated barium microbeads, which also is in accordance with previous findings for Ca^2+^/Ba^2+^ microbeads^23,40^.

We compared the inflammatory potential as detected in the human whole blood assay with the host reactivity’s to the various coated and non-coated alginate microbeads upon implantation into a rodent model. PAV_(high)_ microbeads transplanted into the peritoneal cavity elicited a fibrotic reaction with fibrotic score of ~4.7, while the CHC coating on PAV_(high)_ significantly reduced the fibrosis and cell adhesion score (~2.9). Further on, the low fibrosis provoked by the PAV_(low)_, PAV_(low)_ + CHC and non-coated microbeads was reflected in the whole blood assay showing no activation above the control. The fibrotic scores corresponded well with the outcome in the whole blood assay. In summary, our study shows the *in vivo* biocompatibility outcome to be consistent with the *in vitro* inflammatory responses. This demonstrates the potential of the lepirudin based human whole blood model as a screening tool to evaluate the biocompatibility of surface modified alginate microbeads as an initial test. To our knowledge, this is the first published study directly comparing the biocompatibility of surface modified alginate microbeads in an *in vitro* whole blood model to the fibrotic responses *in vivo*.

If we had succeeded with the surface modifying strategy, the next step would have been the pre-clinical primate model. An objection against this strategy is as we already have demonstrated: the passing in initial testing does not guarantee a success over time in larger animal models^45^. This clearly demonstrates that today’s screening test is not complete, and does not cover all potential factors that could contribute to fibrotic overgrowth. Identifying additional factors could potentially contribute to an improved screening model. The complement system is however an important contributor to initial inflammatory reactions, documented to be involved in a broad range of inflammatory conditions^46^. A recent publication also demonstrated the connection between the complement system and the host response to an implanted material^47^. In accordance with our previous *in vitro* data and the present findings, we conclude that the today’s whole blood model is an efficient screening system, but still not complete. First of all the whole blood model evaluates the initial inflammatory potential which has been shown to be dependent on initial surface complement reactivity^24^. Although this might be a starting point for inflammation, it may not detect all factors, including contribution from other cell types present in the peritoneal cavity, such as fibroblasts, myoblasts and macrophages in adipose tissue. Still, our data points to the whole blood model as an efficient screening assay to reject reactive materials as in the case of PAV_(high)_ coating.

In conclusion, we have demonstrated a new method to bind unique macromolecular heparin conjugates (CHC) to alginate microbeads without losing its biological activity using the cationic linker PAV_(low)_. CHC immobilized alginate microbeads with functional heparin should potentially reduce complement activation and inflammation, thereby preventing PFO and improve graft survival, but would require a less immunogenic cross-linker than PAV. We demonstrated that inflammatory assessments of complement and cytokines using the human whole blood model corresponded to the host response upon implantation in immunocompetent rodents; thus this model could function as an efficient screening tool for designing improved microsphere devices for cell-therapy purposes.

## Methods

### Preparation of alginate microbeads

Alginate microbeads were prepared with ultrapure 2.2% alginate using a stainless steel air-driven droplet generator (Steinau, Berlin, Germany)^18^. The sodium alginate, Pronova UP-MVG (69% guluronic acid, protein content ≤0.3%, endotoxins ≤100 EU/g) was purchased from FMC Biopolymer AS (NovaMatrix, Sandvika, Norway). Briefly, 0.8 ml of 2.2% alginate solution was passed through the air-driven droplet generator with an air flow rate 5 L/min at 100 kPa. The microbeads formed were incubated in a 20 mM barium chloride precipitation bath for 2 min. After gelation, the microbeads were washed thrice in 0.9% NaCl to remove excess barium, and their size counted.

### LBL modification of alginate microbeads

Empty barium alginate microbeads were washed twice in sterile 0.9% saline immediately prior to use. The microbeads were subsequently incubated with polycations PAV, PLL or PLO (3, 10 or 100 µg/ml) in a mass ratio of 1:1 for 15 min at room temperature with gentle mixing. The coated microbeads were washed twice in 0.9% saline to remove excess unbound polycations followed by incubation with CHC (1 mg/ml) in a mass ratio of 2:3 for 30 min at room temperature with gentle mixing, and thereafter washed twice in 0.9% saline to remove excess labelled CHC. For LBL coating of polycations and CHC the above steps were repeated again and a final washing in 0.9% saline was done twice to remove any unbound polyelectrolytes. PAV (9 mg/ml; 55 kDa) and CHC (4.7 mg/ml; >70 kDa) (Patent no: WO2013095270A1) were provided by Corline Systems AB (Uppsala, Sweden).

### Confocal imaging of LBL modified alginate microbeads

The binding of polycations and CHC to alginate microbeads was visualized by confocal laser scanning microscopy (CLSM). CHC (100 µg/ml) was labelled with Alexa 488 (labelling kit from Molecular Probes-Invitrogen, Carlsbad, CA, USA) and PAV with Cy5 (labelling kit from Molecular Probes-Invitrogen), and used at a concentration of either 10 or 100 µg/ml. Images were acquired with a Nikon confocal microscope A1 (Nikon Corporation, Tokyo, Japan) equipped with a Nikon Eclipse inverted Ti microscope stand. Z-stacks of the heparinized microbeads were acquired using the 487 and 561 nm laser line, a 10X objective and 500–550 & 570–620 band pass filters for Alexa 488 and Cy5, respectively. Three-dimensional projections of the acquired Z-stacks were analyzed using NIS-Elements software (Nikon).

### Activated partial thromboplastin time (APTT) assay

The anticoagulant activity of bound CHC on LBL modified microbeads was determined by APTT assay. Briefly, LBL modified and non-coated alginate microbeads were added into an Eppendorf tube containing 500 µL of fresh human plasma. Homogenization of the microbeads was carried out using a glass rod and the resulting plasma supernatant then incubated with 0.1 mL of APTT reagent (TriniCLOT™ aPTT S; Tcoag Ireland Limited) for 2 min at 37 °C. After incubation, 0.1 mL of 0.02 M calcium chloride was added, and the time recorded from this point until the fibrin clot was formed. Both positive (CHC, 1 mg/mL) and negative controls (plasma alone) were used. The APTT assay was carried out in the Haematology Department of the Prince of Wales Hospital, Randwick, New South Wales, Australia.

### Cytotoxicity testing

#### Polycations and heparin conjugate

Mouse fibroblast L929 cells (obtained from the ATCC repository) were seeded at 2 × 10^4^ cells/well (96-well plate) for 24 hr at 37 °C in MEM media (supplemented with 10% FBS, 1% non-essential amino acids, and antibiotics). Thereafter, the conditioned medium was removed, cells washed twice in serum free medium (SFM) before the addition of test samples of the polycationic linkers in SFM (FBS substituted with ITS™ Pre-mix) in concentrations from 0.78–200 μg/ml. For CHC, the stock solution (1000 µg/ml) was serially diluted two-fold to the lowest concentration of 4 µg/ml. Solutions of 5% PBS and 5% DMSO were prepared in SFM and used as negative and positive controls. Samples and controls were incubated with L929 cells (100 µl/well in 4 replicates) for 24 hr, and cell viability measured by MTT (0.5 mg/ml) for the final 4 hr of the culture period. The resultant insoluble formazan product was solubilised with DMSO and absorbance measured at 595 nm. Cell viability was expressed as a percentage of SFM incubated cells. A reduction of cell viability to <70% as compared to SFM control medium alone was regarded as cytotoxic. The cytotoxicity assay was carried out as per ISO 10993-5 “Biological evaluation of medical devices – Part 5: Tests for *in vitro* cytotoxicity”.

#### Direct contact cytotoxicity test of LBL modified alginate microbeads

Mouse fibroblast L929 cells were seeded at 2 × 10^4^ cells/well (96-well plates) for 24 hr at 37 °C in MEM media. Thereafter, medium was removed, cells washed twice with sterile SFM, and then 100 μL of SFM added to each well (n = 3 per sample). A monolayer of LBL modified microbeads (25 microbeads per well) was subsequently added to the surface of L929 cells. After 24 hr, cell viability was measured by MTT. Solutions of 5% PBS and 5% DMSO were prepared in SFM and used as negative and positive controls respectively.

#### Indirect extract cytotoxicity test of LBL modified alginate microbeads

LBL modified microbeads (427 ± 7 per sample) were equilibrated overnight at 37 °C in 2 ml serum free MEM culture media. Thereafter, the supernatant was harvested and cytotoxicity tested by serial dilution. The neat extracts from LBL modified microbeads were assigned as 100%, thereafter a two-fold dilution down to 0.78% v/v of the starting solution was performed, with subsequent cytotoxicity testing.

### Human whole blood assay

A lepirudin based human whole blood assay was used for evaluation of the inflammatory potential^40^. This was approved by the Regional Ethic Committee at NTNU in Norway, and all procedures carried out were in accordance with the relevant guidelines and regulations. Briefly, microspheres were prepared by a dilution protocol^40^ giving ~214 microspheres in a total volume of 100 µl saline. Controls (zymosan 10 µg or saline) were prepared in a total of 100 µl saline. For all samples, 100 µl of PBS with CaCl_2_/MgCl_2_ was added thereafter. Whole blood was drawn from healthy volunteers, with their written informed consent, and 500 µl placed in lepirudin (50 µg/ml) containing vacutained polypropylene vials (4.5 ml). Samples were incubated at 37 °C for 30, 120 or 240 min under continuous rotation, with complement activation stopped by addition of EDTA (10 mM final concentration) followed by centrifugation at 3000 rpm for 15 min. Aliquots of plasma samples were collected and stored at −80 °C for further analysis.

### Quantification of terminal sC5b-9 complex (TCC)

Fluid phase TCC was quantified by a sandwich enzyme linked immunoassay using monoclonal antibodies aE11 (DIA 011-01) specific for soluble sC5b-9 and biotinylated 9C4 (kindly provided by Prof. Mollnes) specific for C6. The assay procedure and the antibody validation have been described previously^48^.

### Quantification of CD11b expression

An activation marker of leucocytes is the CD11b expression, an integrin involved in cell-attachment and increasingly expressed upon activation. CD11b expression was determined after incubating the microbeads for 60 min in lepirudin anti-coagulated human whole blood. Staining for CD11b on both granulocytes and monocytes involved PE anti-CD11b, FITC anti-CD14 and the nuclear dye LDS-751 and analysed using a flow cytometer (Beckman Coulter Epics XL-MCL, Coulter Corp, FL) by measuring MFI^40^.

### Quantification of plasma chemokines, cytokines and growth factors

Quantification of chemokines and cytokines in the plasma was measured with Luminex technology using a Bioplex human cytokine 15-plex cytokine kit (Bio-Rad Laboratories, Hercules, CA). The following 15 inflammatory mediators were analysed: interleukin-1 beta (IL-1β), interleukin-10 (IL-10), interleukin-6 (IL-6), interleukin-1 receptor antagonist (IL-1Ra), tumour necrosis factor (TNF), interferon gamma (IFN-γ), macrophage migration inhibitory factor (MIF), interleukin-8 (IL-8), macrophage inflammatory protein (MIP-1α), monocyte chemoattraction protein-1 (MCP-1), interferon gamma-induced protein 10 (IP-10) and regulated upon activation T-cell expressed and secreted (RANTES), platelet derived growth factor-BB (PDGF-BB), vascular endothelial growth factor (VEGF) and hepatocyte growth factor (HGF). In the last part, IL-8 and TNF was quantified by ELISA (R&D Systems, MN, USA).

### Implantation of LBL modified alginate microbeads

For testing the *in vivo* biocompatibility, LBL modified or non-modified microbeads were transplanted into the peritoneal cavity of immunocompetent rats^18^, as approved by the Animal Care and Ethics Committee of CSIRO, North Ryde, Australia. All experiments with animals were performed in accordance with the CSIRO guidelines and regulations. Briefly, female Wistar rats (4–5 weeks) were anaesthetized with inhalational isoflurane, and LBL modified microbeads with a packed cell volume of 0.7 ml delivered into the peritoneal cavity in a volume of 1–2 ml PBS through a 14 gauge catheter via a ventral midline incision. The transplanted rats were euthanized at three weeks post-implantation and the graft assessed for fibrotic overgrowth.

### Graft retrieval and evaluation of pericapsular fibrosis

LBL modified and non-modified microbeads were recovered from freshly euthanized animals by peritoneal lavage with sterile PBS, and separated from the peritoneal lavage fluid by sedimentation. The peritoneal cavity was then flushed with sterile PBS and subsequently examined for any remaining microbeads. The following were assessed i) retrieval rate ii) state of retrieved microbeads iii) evidence of breakage (%) and iv) fibrotic overgrowth^18^.

### Statistical analysis

All data are presented as mean ± standard error of mean (SEM). Differences between two groups were analysed by the two-tailed Student’s t-test and if more than two groups by one-way ANOVA with *post-hoc* Duncan’s Multiple-Comparison test. The software NCSS 2004 (NCSS, Kaysville, UT) was used to perform the statistical data analysis. Significant differences among data groups were assigned when *p* < 0.05.

### Data availability

The datasets generated during and/or analysed during the current study are available from author V.V. on reasonable request.

## Electronic supplementary material

Supplementary information
