# Supplementary material for: In Vitro and In Vivo Biocompatibility Evaluation of Polyallylamine and Macromolecular Heparin Conjugates Modified Alginate Microbeads
Source: Sci Rep. 2017 Sep 15;7:11695. doi: 10.1038/s41598-017-11989-1 (PMC5600981; doi:10.1038/s41598-017-11989-1)

In Vitro and *In Vivo* Biocompatibility Evaluation of Polyallylamine and Macromolecular Heparin Conjugates Modified Alginate Microbeads

Vijayaganapathy Vaithilingam[a], Bjørg Steinkjer[b], Liv Ryan[b], Rolf Larsson[c,d], Bernard Edward Tuch[a,e*], Jose Oberholzer[f], Anne Mari Rokstad[b,g]

[a] Materials Science and Engineering, Commonwealth Scientific and Industrial Research Organization (CSIRO), North Ryde, New South Wales, Australia

[b] Centre of Molecular Inflammation Research and Department of Cancer Research and Molecular Medicine, Norwegian University of Science and Technology (NTNU), Trondheim, Norway

[c] Corline System AB, Uppsala, Sweden

[d] Department of Immunology, Genetics and Pathology, University of Uppsala, Uppsala, Sweden

[e] School of Medical Sciences, University of Sydney, Sydney, New South Wales, Australia

[f] Department of Surgery, University of Illinois at Chicago, Chicago, Illinois, United States of America

[g] The Central Norway Regional Health Authority (RHA), Trondheim, Norway

*Corresponding author

Dr. Bernard Tuch
PO Box 821
Maroubra, NSW 2035
Australia
Email: bernard.tuch@uni.sydney.edu.au
Tel: +61 411 461 604
Fax: +61 2 9389 6656

Supplementary Information

Appendix. Cytotoxicity of cationic linkers and heparin conjugate. The cytotoxicity of three polycationic linkers PAV, PLL and PLO and CHC were tested using the mouse fibroblast L929 cells. PLL and PLO were both cytotoxic at concentrations ≥ 12.5 µg/ml with cell viabilities below the cut-off value of 70% (Supplementary figure 2A). The morphology of L929 cells was significantly affected at higher concentrations with the cells appearing rounded and shrunken similar to 5% DMSO positive control (Supplementary figure 3). At concentrations < 12.5 µg/ml both PLL and PLO were non-toxic with cells looking healthy and morphology similar to L929 cells cultured in SFM reference or 5% PBS negative controls. PAV, on the other hand, was cytotoxic up to concentrations of 6.25 µg/ml and cell morphology did not return to normal until the concentration was 3.125 µg/ml (Supplementary figures 2A&3). At concentrations < 3.125 µg/ml, PAV was non-toxic with cell viabilities above the cut-off value of 70% with healthy and normal morphology. The CHC was not cytotoxic at any of the dilutions tested with only a slight reduction in viability to ~71% at the highest concentration of 1 mg/ml (Supplementary figure 2B). No changes in cell morphology could be detected at any concentrations (Supplementary figure 3).

Supplementary figure 1. Confocal imaging of LBL modified microbeads. Representative confocal images of LBL modified alginate microbeads containing high (100 µg/ml) or low (10 µg/ml) concentrations of fluorescently labelled PAV (PAV-Cy5.5; red) and 36 µg/ml CHC (CHC-Alexa 488; green) cultured for 1, 7, 14 and 21 days in phosphate buffered saline post-heparinization. The figures are representative confocal images of LBL modified nicrobeads taken at day 1 post-heparinization. Arrows point to intermittent thick patches of PAV exposed without sufficient masking by CHC as seen in higher magnification images of $PAV_{(high)}$+CHC microbeads.

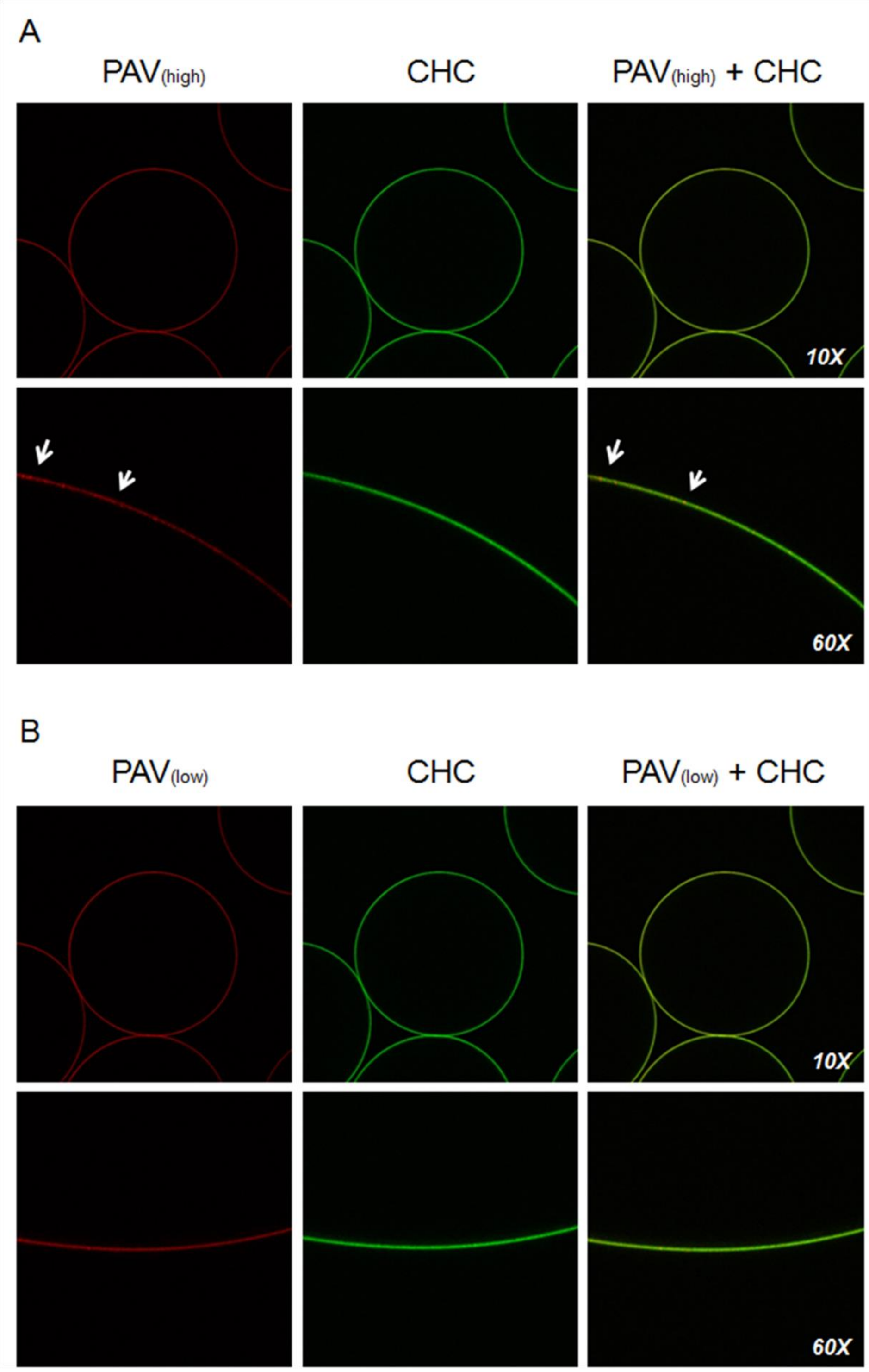

Supplementary figure 2. Cytoxicity of the polycationic linkers. Serial dilution cytotoxicity assay for polycationic linkers PAV, PLL & PLO (A) and macromolecular CHC (B) using mouse fibroblast L929 cells. A cut-off value of < 70% is deemed cytotoxic as seen with the positive control 5% DMSO and a value > 70% is non-toxic as seen with negative control 5% PBS and SFM reference control. Values = mean ± SEM (n=3 for each polycationic linkers and CHC).

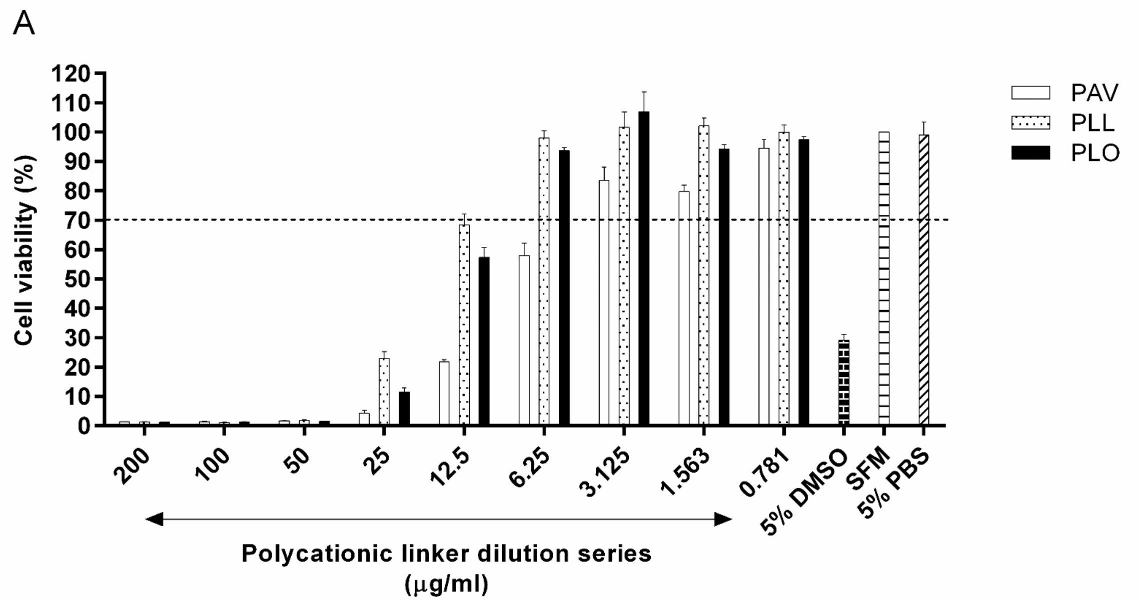

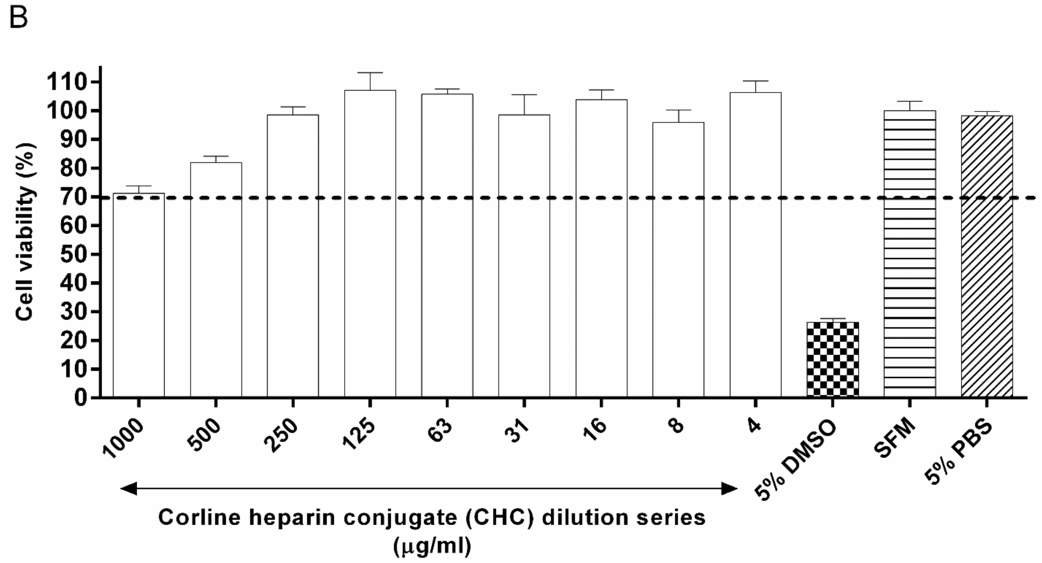

Supplementary figure 3. Morphology of L929 cells. Representative morphology pictures of L929 cells exposed to various concentrations of polycationic linkers PAV, PLL and PLO and the macromolecular CHC compared to controls 5% DMSO, SFM and 5% PBS after 24 hr in culture.

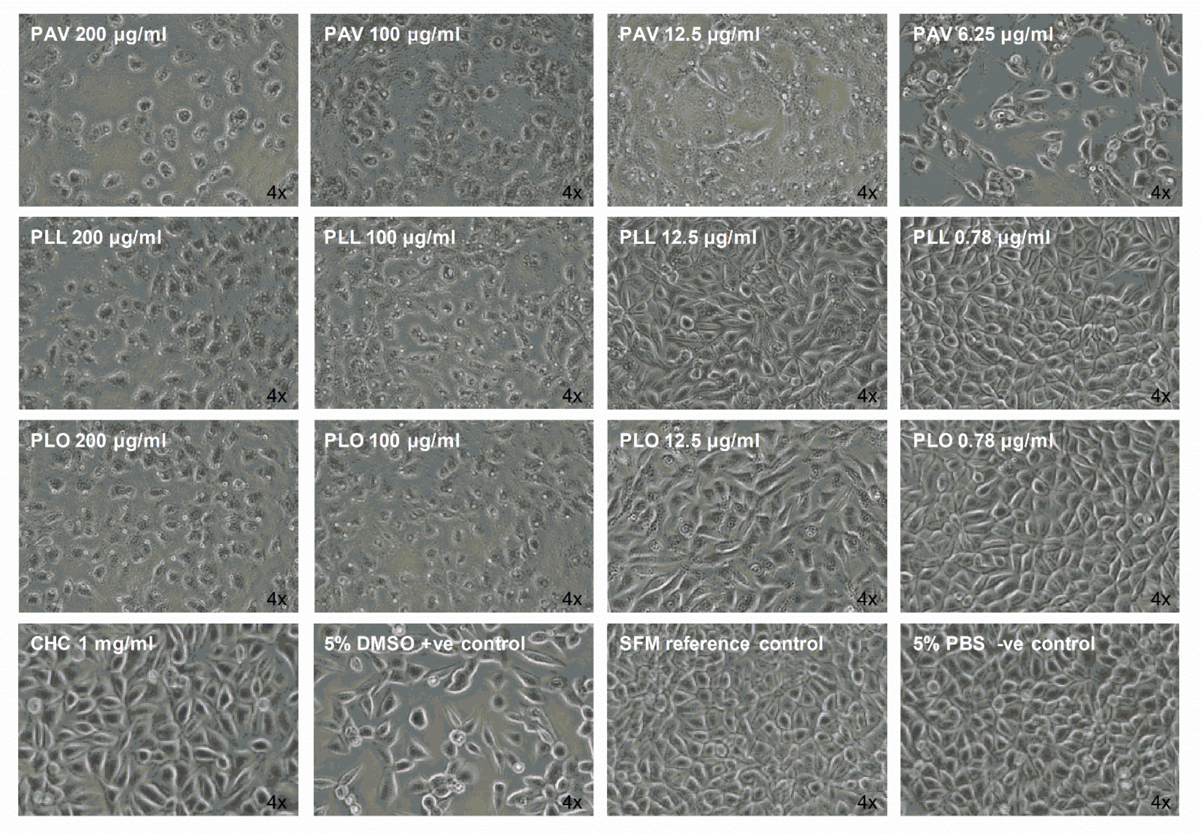

Supplementary figure 4. Effect of LBL modified microbeads on the kinetics of TCC formation. Time-dependent TCC formation in human blood after addition of $PAV_{(high)}$ (A) and $PAV_{(low)}$ (B) containing LBL modified microbeads with/without CHC compared to positive control zymosan. TCC formation significantly ($p<0.0001$) increased with time with the addition of saline, non-coated and surface modified alginate microbeads (ANOVA with *posthoc* Duncan's Multiple-Comparison test). Values are mean ± SEM and obtained from two separate studies. For the first study $PAV_{(high)}$ and $PAV_{(high)}$+CHC microbeads were incubated in whole blood obtained from 5 different donors (A). For the next study $PAV_{(low)}$ and $PAV_{(low)}$+CHC microbeads were incubated in whole blood obtained from 4 different donors (B).

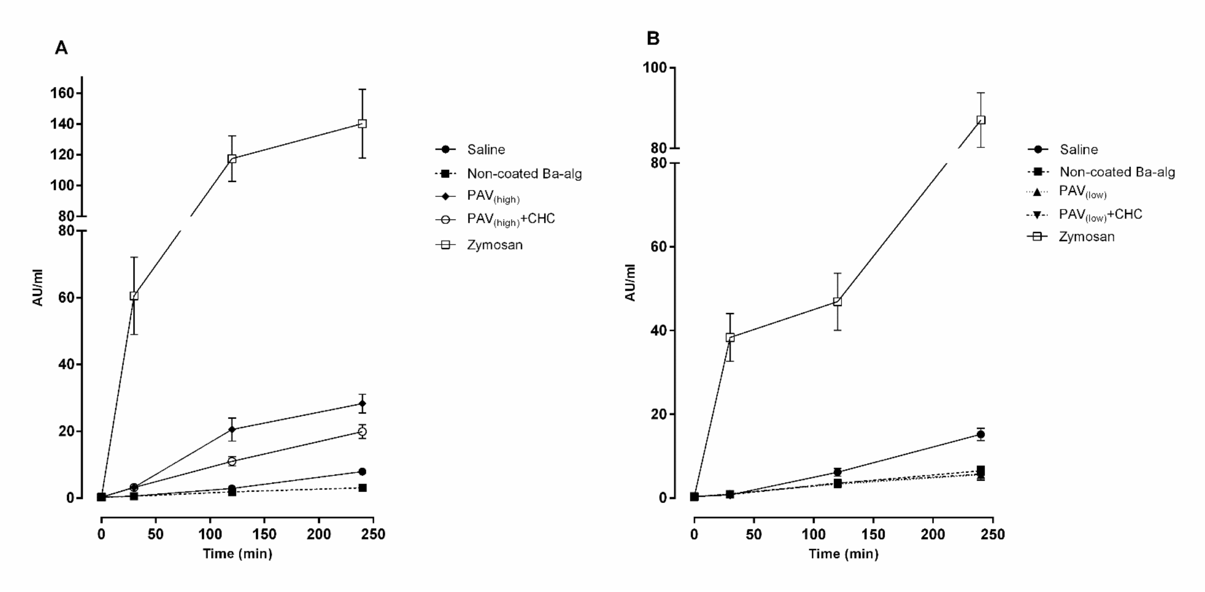

Supplementary figure 5. Effect of LBL modified microbeads on leukocyte activation. Leukocyte activation as measured by CD11b expression on granulocytes (A&C) and monocytes (B&D) after incubation of whole blood with saline, non-coated and varied coated alginate microbeads for 240 min. Values (measured as MFI) are mean ± SEM from two separate studies. For the first study $PAV_{(high)}$ and $PAV_{(high)}$+CHC microbeads were incubated in whole blood obtained from three different donors (A&B). Plasma baseline values (MFI) for granulocyte and monocyte CD11b expression measured at the start of the study were 50.6 ± 4.7 and 107.3 ± 7.6 respectively, and positive control zymosan values (MFI) were 830.4 ± 81.1 and 851.7 ± 31.5 for granulocyte and monocyte respectively. For the second study $PAV_{(low)}$ and $PAV_{(low)}$+CHC microbeads were incubated in whole blood obtained from three different donors (C&D). Plasma baseline values (MFI) for granulocyte and monocyte CD11b expression measured at the start of the study were 108.4 ± 47.6 and 139.3 ± 51.1 respectively, and positive control zymosan values (MFI) were 1318 ± 78.8 and 992.3 ± 66.2 for granulocyte and monocyte respectively.

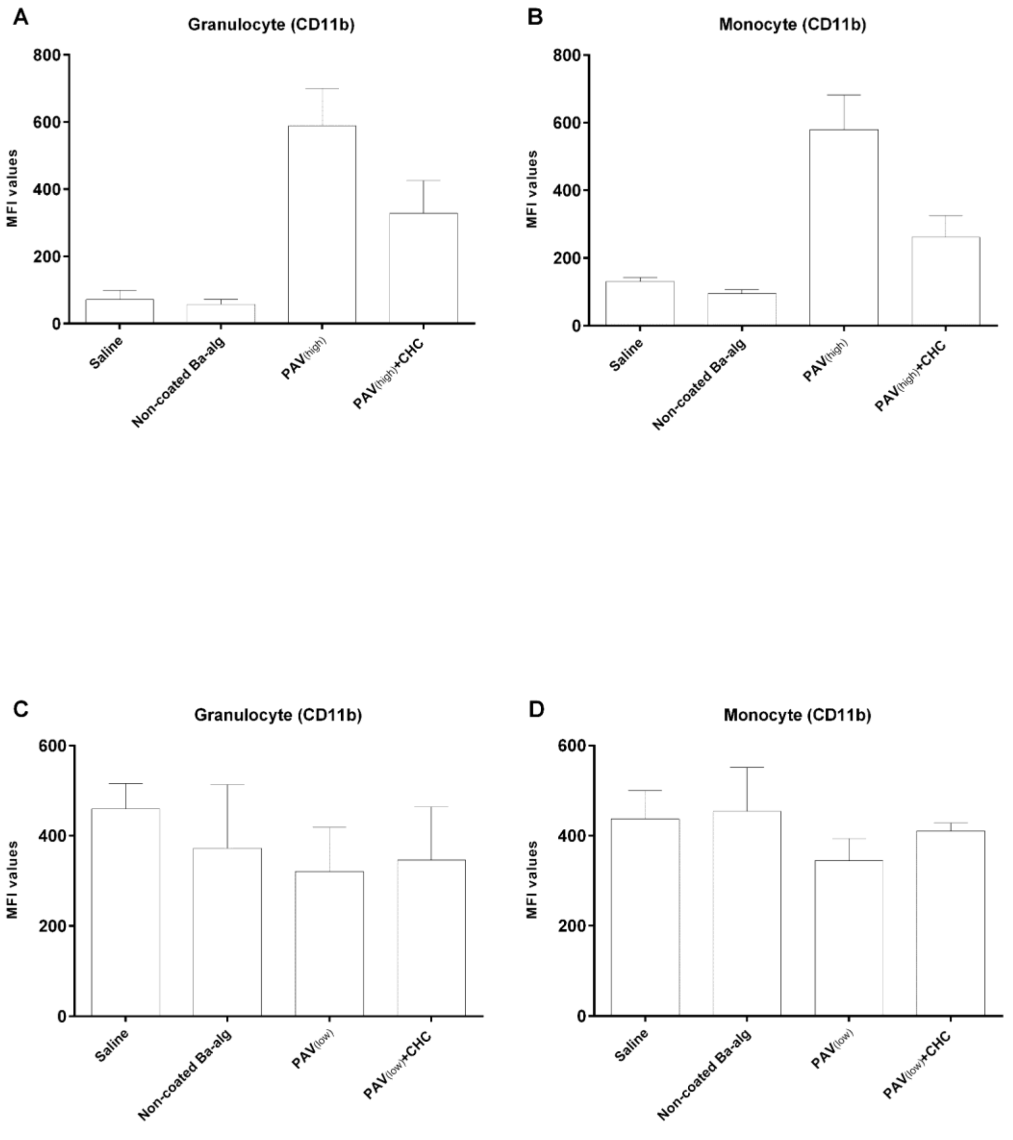

Supplementary figure 6. Effect of LBL modified microbeads on TNF-α and IL-8. $PAV_{(high)}$, $PAV_{(high)}$+CHC, $PAV_{(low)}$, $PAV_{(low)}$+CHC and non-coated Ba-alg microbeads were incubated in lepirudin anti-coagulated whole blood and inflammatory mediators measured in plasma after 240 min. Values are mean ± SEM and measured in plasma obtained from four to five independent donors. (A) The plasma baseline and positive control values (pg/ml) for TNF-α 2.4 ± 1.7 (baseline) and 17346 ± 3575 (zymosan); IL-8 1.7 ± 1.6 (baseline) and 6240 ± 691.7 (zymosan). (B) The plasma baseline and positive control values (pg/ml) for TNF-α 44.03 ± 7.9 (baseline) and 1815 ± 398.5 (zymosan); IL-8 69 ± 2.4 (baseline) and 4593 ± 825.1 (zymosan).

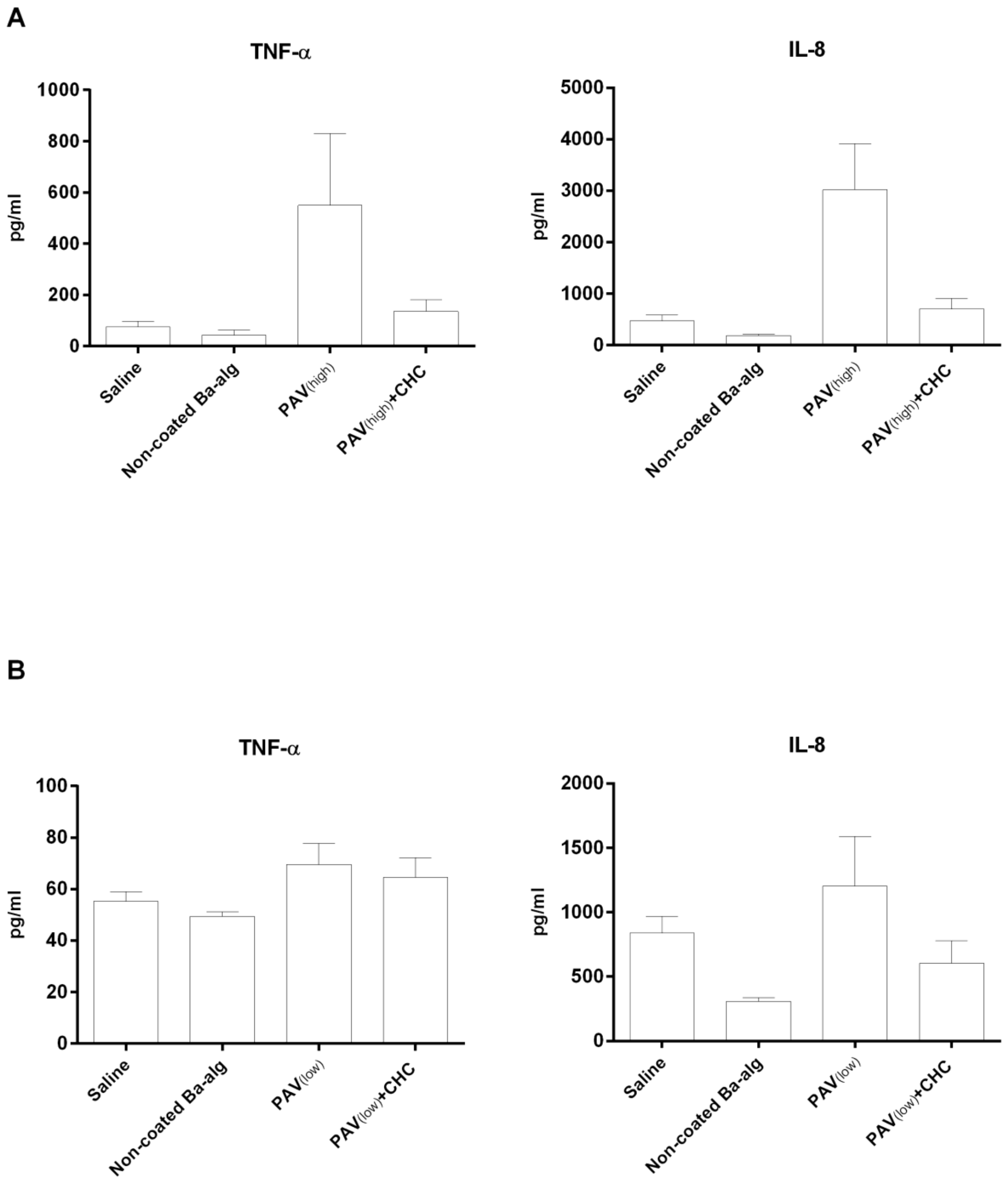

Supplementary figure 7. Effect of $PAV_{(high)}$ containing LBL modified microbeads on inflammatory mediators. $PAV_{(high)}$, $PAV_{(high)}$+CHC and non-coated Ba-alg microbeads were incubated in lepirudin anti-coagulated whole blood and inflammatory mediators measured in plasma after 240 min by Bioplex. Values are mean ± SEM and measured in plasma obtained from five independent donors. The plasma baseline and positive control values (pg/ml) for IL-1β < 0.11 (baseline) and 2290 ± 516.3 (zymosan), IL-6 0.71 ± 0.3 (baseline) and 7119 ± 416.7 (zymosan), MIP-1α 2.44 ± 0.07 (baseline) and 3027 ± 542.9 (zymosan), MCP-1 45.01 ± 3.9 (baseline) and 124.6 ± 13.2 (zymosan), IL-1Ra 88.8 ± 36.3 (baseline) and 1428 ± 326.2 (zymosan), IL-10 0.8 ± 0.3 (baseline) and 5.4 ± 0.5 (zymosan), MIF 527.5 ± 225.9 (baseline) and 2117 ± 516.5 (zymosan), VEGF 5.5 ± 1.2 (baseline) and 38.02 ± 15.2 (zymosan), HGF below detectable limit (baseline) and 882.3 ± 204.5 (zymosan). IP-10 217.3 ± 61.5 (baseline) and 1169 ± 370.2 (zymosan), RANTES 2042 ± 838.1 (baseline) and 1299 ± 495.1 (zymosan), PDGF-BB 90.2 ± 28.4 (baseline) and 589 ± 188.6 (zymosan).

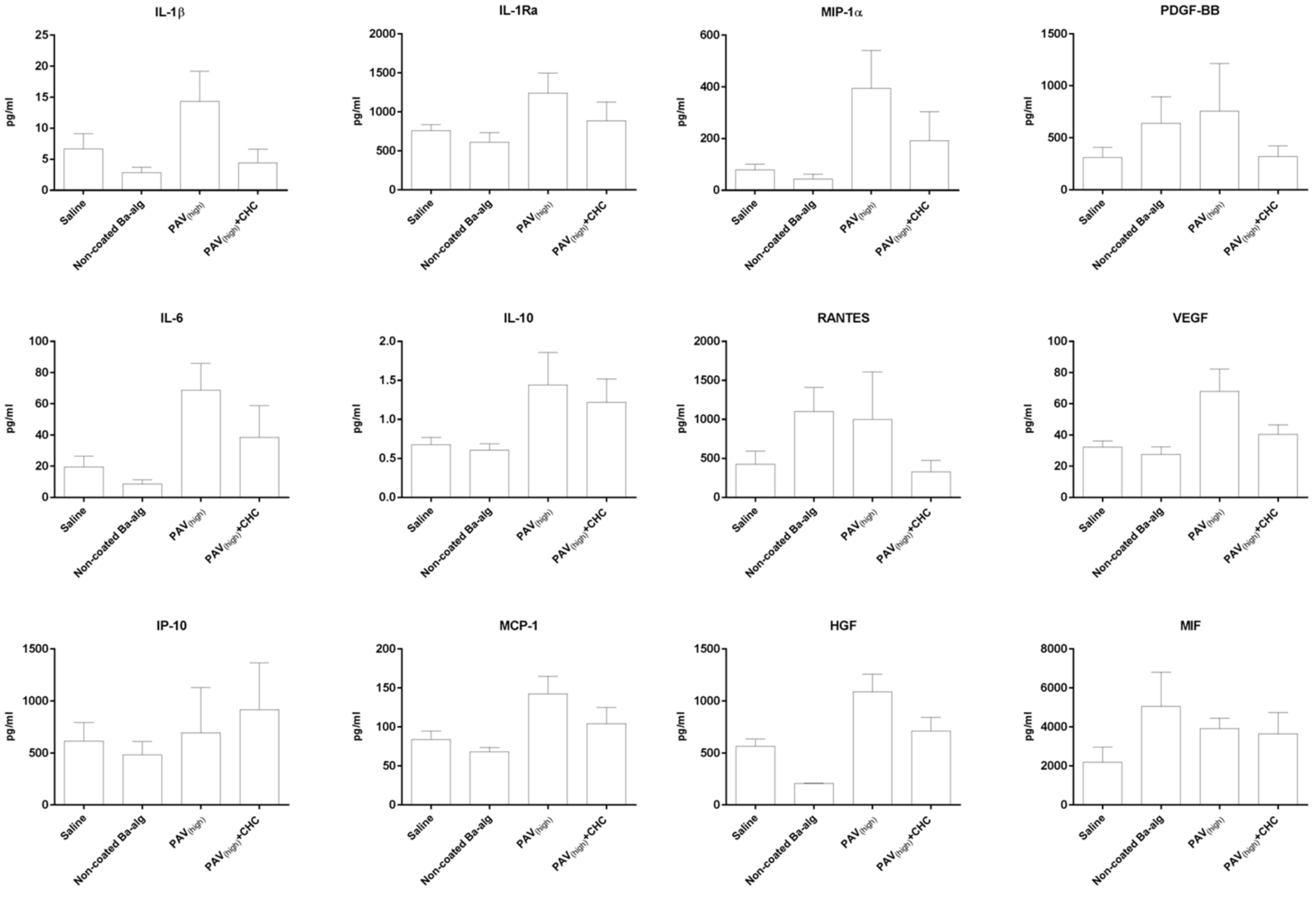

Supplement: Supplementary file 1 — Supplementary information [file 41598_2017_11989_MOESM1_ESM.pdf]
